# Insights on the Structural Variations of the Furin-Like Cleavage Site Found Among the December 2019–July 2020 SARS-CoV-2 Spike Glycoprotein: A Computational Study Linking Viral Evolution and Infection

**DOI:** 10.3389/fmed.2021.613412

**Published:** 2021-03-10

**Authors:** Marni E. Cueno, Miu Ueno, Rinako Iguchi, Tsubasa Harada, Yoshifumi Miki, Kanae Yasumaru, Natsumi Kiso, Kanta Wada, Koki Baba, Kenichi Imai

**Affiliations:** ^1^Department of Microbiology, Nihon University School of Dentistry, Tokyo, Japan; ^2^Immersion Biology Class, Department of Science, Tokyo Gakugei University International Secondary School, Tokyo, Japan; ^3^Immersion Physics Class, Department of Science, Tokyo Gakugei University International Secondary School, Tokyo, Japan

**Keywords:** furin-like cleavage site, infection clusters, SARS-CoV-2 (SARS2), spike glycoprotein, structural variations

## Abstract

The SARS-CoV-2 (SARS2) is the cause of the coronavirus disease 2019 (COVID-19) pandemic. One unique structural feature of the SARS2 spike protein is the presence of a furin-like cleavage site (FLC) which is associated with both viral pathogenesis and host tropism. Specifically, SARS2 spike protein binds to the host ACE-2 receptor which in-turn is cleaved by furin proteases at the FLC site, suggesting that SARS2 FLC structural variations may have an impact on viral infectivity. However, this has not yet been fully elucidated. This study designed and analyzed a COVID-19 genomic epidemiology network for December 2019 to July 2020, and subsequently generated and analyzed representative SARS2 spike protein models from significant node clusters within the network. To distinguish possible structural variations, a model quality assessment was performed before further protein model analyses and superimposition of the protein models, particularly in both the receptor-binding domain (RBD) and FLC. Mutant spike models were generated with the unique ^681^PRRA^684^ amino acid sequence found within the deleted FLC. We found 9 SARS2 FLC structural patterns that could potentially correspond to nine node clusters encompassing various countries found within the COVID-19 genomic epidemiology network. Similarly, we associated this with the rapid evolution of the SARS2 genome. Furthermore, we observed that either in the presence or absence of the unique ^681^PRRA^684^ amino acid sequence no structural changes occurred within the SARS2 RBD, which we believe would mean that the SARS2 FLC has no structural influence on SARS2 RBD and may explain why host tropism was maintained.

## Introduction

Coronaviruses (CoV) are enveloped positive-stranded RNA viruses that have the largest genome among all known RNA viruses and, at present, there are seven known CoVs capable of infecting humans (1–7). Among CoV structural proteins, the spike protein is a class I viral fusion protein that is involved in viral entry, host tropism determination, viral pathogenesis, and host immune response induction (8–11). The spike protein is comprised of three segments (large ectodomain, single-pass transmembrane anchor, and short intracellular tail) (11), with the ectodomain further divided into the S1 receptor-binding subunit and S2 membrane-fusion subunit (10, 11). During a typical CoV infection, S1 binds to an ideal host receptor enabling viral attachment and, consequently, S2 would fuse the host and viral membranes, allowing viral genetic material to enter host cells (10, 11).

Interestingly, prior to SARS-CoV-2 (SARS2), there were six human pathogenic coronaviruses (10), with SARS2 resulting in a pandemic causing the coronavirus disease 2019 (COVID-19) (12, 13). With regards to the homotrimeric spike protein, the SARS2 spike protein follows the same mechanism of viral entry used by SARS-CoV-1, wherein, the SARS2 spike protein binds to a functional receptor human angiotensin-converting enzyme 2 (ACE2) via the 6-residue (L455, F486, Q493, S494, N501, Y505) receptor-binding domain (RBD) (10, 14). One notable structural feature of the SARS2 spike protein is the presence of a polybasic (furin-like) cleavage site (^682^RRAR^685^) which has been found to be disordered (15, 16) and, likewise, linked to effective furin cleavage that could help determine viral pathogenesis and host tropism (17–19). Moreover, the comparative analysis of the intrinsic disorder predisposition of spike protein from SARS2, SARS, and Bat CoV revealed that the furin-like cleavage site of SARS spike is incorporated within the longer disordered region ^676^TQTNSPRRARSVAS^691^, which is not present in spike proteins from SARS and Bat CoV (20). The presence of disorder in a region containing a polybasic (furin-like) cleavage site is an extremely important point, as an intrinsic disorder at the cleavage site is crucial for efficient protease action (20, 21). Furthermore, aside from the presence of the polybasic cleavage site (^682^RRAR^685^), SARS2 likewise has an inserted leading proline (P681), which is suggested to improve protease active site accessibility not only by furin proteases but other proteases as well (21). Thus, this would mean that the inserted sequence unique for SARS2 is the ^681^PRRA^684^ sequence (18).

The structural orientation of either individual or a series of amino acids plays an important role in establishing both protein configuration and protein-protein complexes (22), which likewise may affect protein function (23). This would imply that any probable changes in structural orientation occurring in the SARS2 spike furin-like cleavage (including P681) site (FLC) may have an impact on viral infectivity (24). However, to our knowledge, this has never been fully elucidated. A better understanding of the potential effects of the structural orientation changes occurring within the SARS2 FLC site may shed light on the occurrence of varying SARS2 variants and, more importantly, its role in viral reinfection, potentially leading to novel drug design and therapeutic strategies.

## Materials and Methods

### COVID-19 Genomic Epidemiology Network Design and Analyses Between December 2019 and July 2020

Network analyses were performed in order to gather a holistic understanding of the phylogeny of the COVID-19 genomic epidemiology (25). For this study, network design followed the phylogenetic tree of the COVID-19 genomic epidemiology, based on the GISAID website (www.gisaid.org) between December 2019 and July 2020. A total of 2,793 genomes were used for both network design and analyses. We used Cytoscape for both network design and analyses (26). For network design, nodes were made to represent the countries (indicated as a box) and phylogenetic branch points (indicated as dots) while the edges represent the phylogenetic lineage originating from either a country or branch point. For network analyses, the following centrality measurements were initially analyzed: (1) stress centrality (identifying important nodes); (2) eccentricity centrality (identifying accessible nodes); (3) closeness centrality (identifying relevant nodes); (4) betweenness centrality (identifying crucial nodes); and (5) edge betweenness centrality (identifying significant edges) (27). Briefly, nodes (Supplementary Figure 1) and edges (Supplementary Figure 2) above a computed threshold for each centrality were considered significant. A unified network was designed based on all centrality measurements used for this study (both nodal and edge centralities) and, more importantly, nodes that were linked to either nodes or edges that are above the threshold based on all five centrality measurements used were determined.

### SARS2 Spike Protein Modeling

Representative SARS2 spike amino acid sequences (*n* = 263) deposited between December 2019 and July 2020 were collected from the National Center for Biological Information (NCBI). The selection of sequences was based on the results obtained from our previous COVID-19 genomic epidemiology network analyses. Moreover, representative monomeric SARS2 spike models were selected using Tm align (28). Briefly, a minimum of 10 generated sequence models were initially obtained. Further structural analyses used spike models with similar Root Mean Square Deviation (RMSD) values and Template Modeling scores (Tm-scores) based on superimposition. In particular, the SARS2 spike models used for further structural analyses were based on structural variations in SARS2 FLC and have the following Genebank accession numbers: MT019529, MN994468, MT020781, MT825091, MT467261, MT658503, MT499218, MT549887, and MT461625. The Phyre2 web server (29) was used to generate all protein models while the Jmol applet (30) was used for protein visualization.

### Protein Model Quality Assessment

To confirm the accuracy and suitability of the generated SARS2 spike protein models for further analyses, both contact mapping and protein model:crystal structure superimposition were performed for model quality assessment. A protein contact map was made using the CMView applet to determine the common contact between the model and crystal (31). Moreover, higher common contact (>90%) would mean more structural similarities (32), which would mean that the generated model is suitable for further analyses. Subsequently, representative SARS2 spike cryo-EM structure (PDB ID: 6XR8) (15) and a monomeric 6XR8 model (cryo-EM model) generated using Phyre 2 were used for superimposition (using Tm align) to serve as a model quality check. For this study, SARS2 spike models were considered suitable for further analyses if superimposed sequence model:crystal and crystal model:crystal have RMSD < 1.50.

### Comparison of SARS2 Spike Models

All structural comparisons conducted focused on both the SARS2 FLC and RBD. Moreover, two sets of structural comparisons were made. The first set of structural comparisons focused on contrasting the SARS2 FLC and RBD among all representative SARS2 spike models through superimposition. One of the representative models (generated from MT019529) was used as the common model for superimposition. The second set of structural comparisons involved producing mutants from all representative SARS2 spike models without the ^681^PRRA^684^ sequence unique in SARS2. A protein threading approach (via Phyre 2) was used to generate the mutant models. Similarly, focusing on SARS2 FLC and RBD, the original model (with ^681^PRRA^684^) was compared to the mutated model (without ^681^PRRA^684^) through superimposition using Tm align. Model superimposition (focusing on SARS2 FLC and RBD), RMSD values, and Tm scores were established using Jmol and Tm align, respectively.

## Results

### Nine Node Clusters From the COVID-19 Genomic Epidemiology Network Were Established Between December 2019 and July 2020

**Figure 1:**
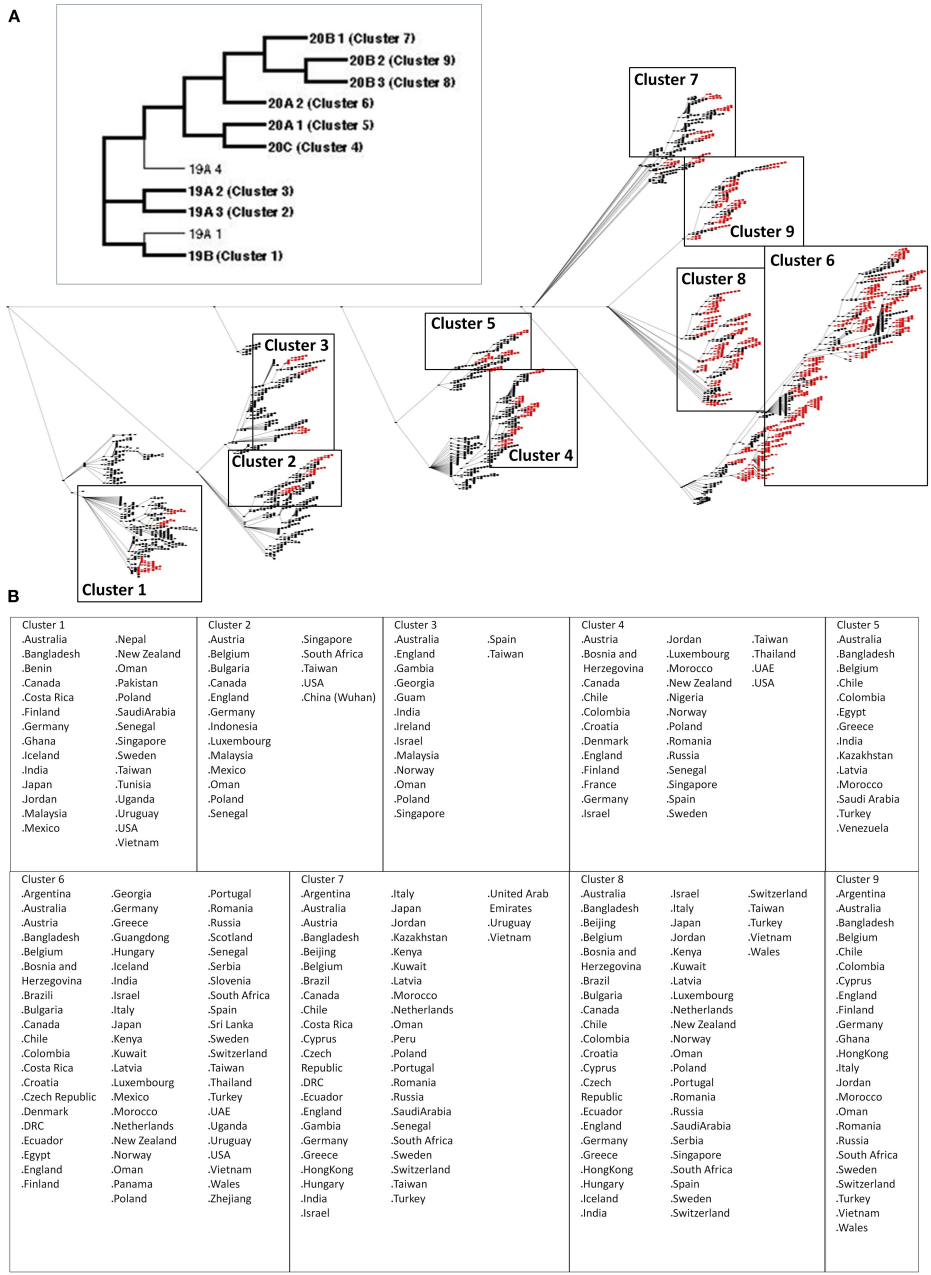
Nine significant node clusters within the COVID-19 genomic epidemiology network designed between December 2019 and July 2020. **(A)** COVID-19 genomic epidemiology network. (Upper panel) Simplified network, with the genomic clades and node clusters labeled. (Lower panel) Actual network, with the significant nodes (red) as determined by centrality analyses are shown. Nodes (dots) and edges (lines) are indicated. Node clusters are boxed and labeled. **(B)** List of countries identified by the significant nodes and classified according to node cluster.

The SARS2 genome is constantly evolving, and genome distribution varies in terms of geographic location (33, 34). To establish possible node clusters within the COVID-19 genomic epidemiology network established between December 2019 and July 2020, network analytics was performed to elucidate the holistic and simultaneous analyses of complementary data (27, 35). One of the key points of network analytics is centrality analysis, which involves collecting network components in order to distinguish important elements and, likewise, requires several centrality measurements to be considered fully efficient for analyzing networks (27, 36). Considering this and the five different centrality measurements used to identify node clusters, this would suggest that the results obtained are reliable. Interestingly, we were able to identify nine node clusters, encompassing various SARS2 genomic clades classified by the GISAID website (Figure 1A). We observed that some of the countries identified among the nine node clusters are likewise found in other node clusters (regardless of belonging to different SARS2 clades) (Figure 1B). These results could mean that the putative significant node clusters are not dependent on SARS2 clades, which coincidentally are based on viral genome mutations (34). This insinuates that there could be other similarities among the node clusters with regard to SARS2 pathogenesis. Considering that the SARS2 FLC is crucial for viral pathogenesis and host tropism (17–19), which we believe would imply that the SARS2 FLC is a conserved structural feature (18), we postulate that the SARS2 FLC could be a common structural feature among the node clusters. We wish to emphasize that our current study mainly focused on the SARS2 FLC structural feature. In possible future work, it would be interesting to recognize other possible spike protein structural features found among the node clusters identified.

### SARS2 Spike Models Are Suitable for Structural Analyses

**Figure 2:**
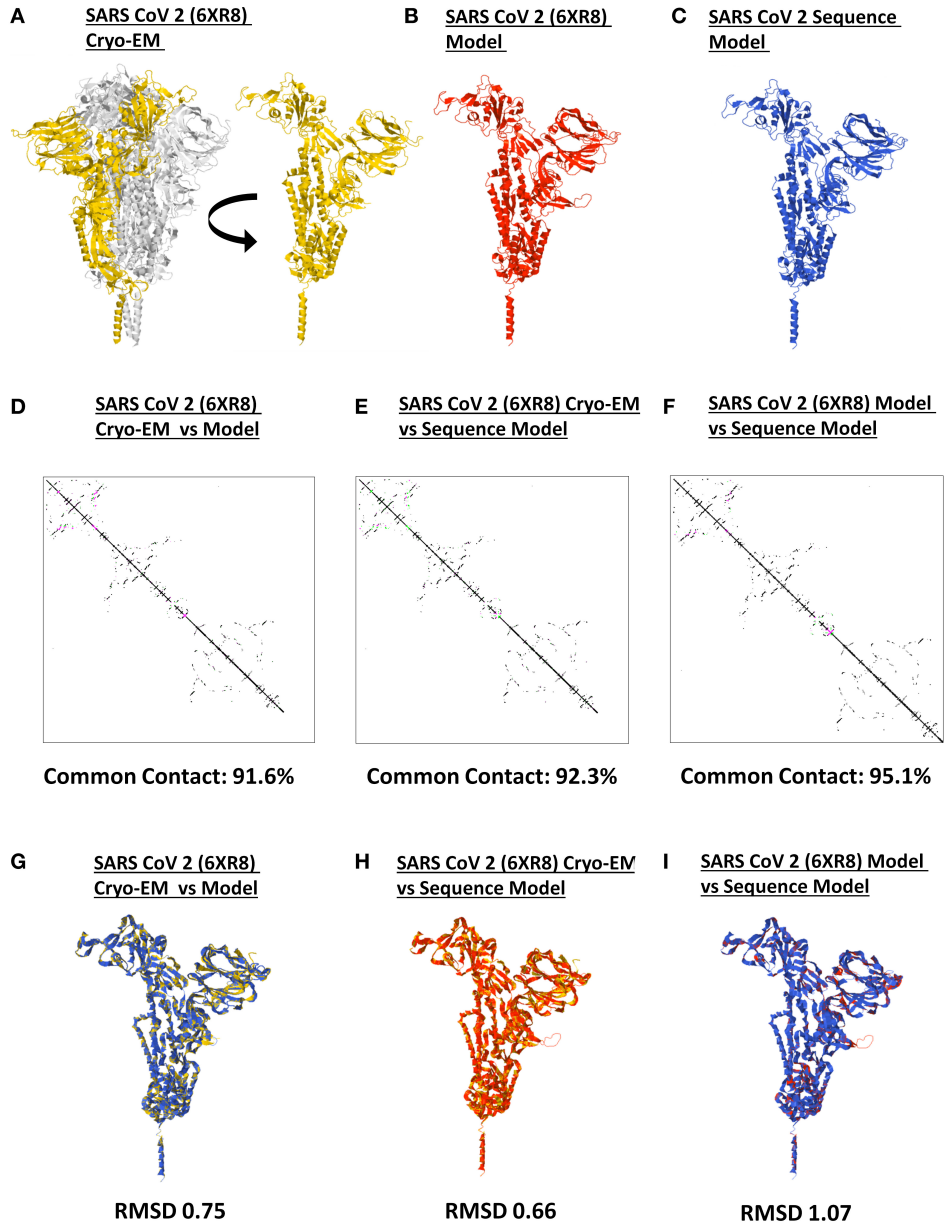
Model quality assessment of a generated monomeric SARS-CoV-2 spike protein. Representative SARS-CoV-2 **(A)** 6XR8 cryo-EM, **(B)** 6XR8 model, and **(C)** sequence model of monomeric spike proteins are indicated. Contact maps of **(D)** 6XR8 cryo-EM and model, **(E)** 6XR8 cryo-EM and sequence model, and **(F)** 6XR8 model and sequence models are shown. The common contact of the protein structures being compared is labeled below. Superimposition between **(G)** 6XR8 cryo-EM and model, **(H)** 6XR8 cryo-EM and sequence model, and **(I)** 6XR8 model and sequence models are presented. RMSD scores of the superimposed protein structures are indicated below. SARS CoV 2 6XR8 cryo-EM (yellow), 6XR8 model (red), and sequence model (royal blue) are indicated.

It has long been recommended that model quality assessment be performed prior to any downstream structural analyses using protein structures generated from either experimental (i.e., crystallized) or theoretical (i.e., computer-based) methods (37). To establish the reliability and suitability of all SARS2 spike models generated, both protein contact maps and structural superimpositions were performed. Representative SARS2 crystal structure (Figure 2A), SARS2 crystal model (Figure 2B), and SARS2 sequence model (Figure 2C) were used for all superimpositions conducted. We observed that protein contact map superimposition between crystal model:crystal structure (Figure 2D), sequence model:crystal structure (Figure 2E), and sequence model:crystal model (Figure 2F) have high common contact (>90%), which implies that there is high contact similarity between the superimposed structures. We only considered SARS2 spike monomers when examining structural superimpositions. We also observed that RMSD values between cryo-EM model:crystal structure [RMSD 0.75] (Figure 2G), sequence model:cryo-EM structure [RMSD 0.66] (Figure 2H), and sequence model:cryo-EM model [RMSD 1.07] (Figure 2I) were RMSD < 1.5 which in-turn were considered adequate for further analyses (38). These results (both protein contact map and structural superimpositions) would suggest that the generated SARS2 spike models are suitable for further structural analyses.

### Nine SARS2 FLC Structural Patterns Were Identified Among the Nine Node Clusters

Protein structure and conformation dynamics have often been correlated to biological function, which emphasizes the importance of protein structural pattern variations (23). To elucidate the possible SARS2 FLC structural variations among the 9 node clusters, representative SARS2 models from each node cluster were superimposed with the SARS2 model generated from MT019529 (Wuhan, China) as a comparison. Since SARS2 FLC also affects host tropism, SARS2 RBD was similarly checked.

**Figure 3:**
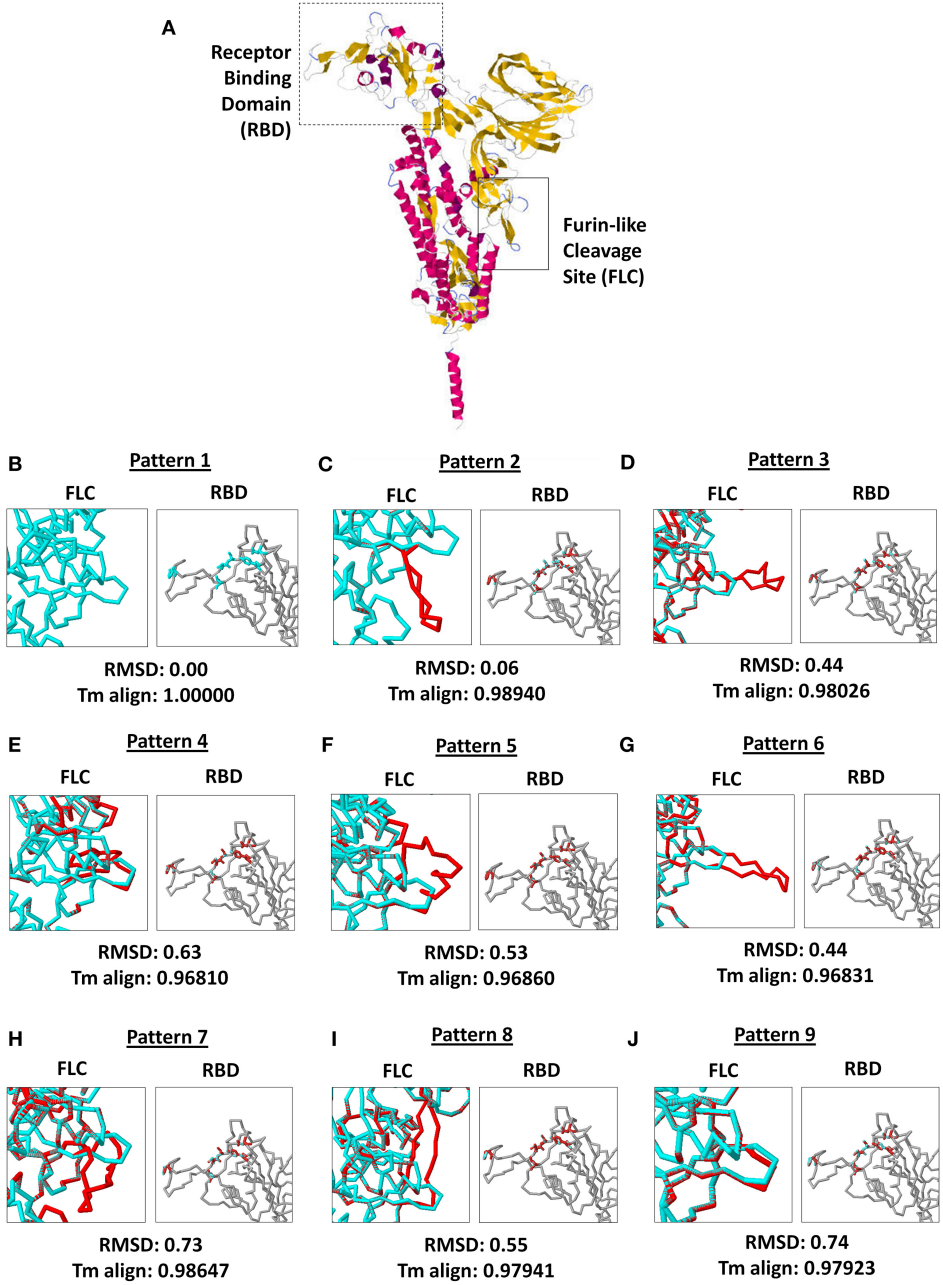
Comparison of the 9 SARS-CoV-2 spike protein furin-like cleavage site structural patterns and corresponding receptor binding domains. **(A)** Representative monomeric SARS-CoV-2 spike protein model with the receptor binding domain (boxed dash lines) and furin-like cleavage site (boxed solid lines) indicated. **(B–J)** Superimposed spike protein models showing the nine structural patterns of the furin-like cleavage site (*left panel*) and receptor binding domain (*right panel*). Pattern 1 SARS-CoV-2 spike protein model (cyan) and the eight other structural patterns (red) are shown. RMSD scores and Tm align values normalized to Pattern 1 SARS-CoV-2 spike protein model are indicated below.

As seen in Figure 3A, both SARS2 RBD (box dash lines) and FLC (box solid lines) structural changes were the focus of the study. Interestingly, we found nine SARS2 FLC structural patterns (Figures 3B–J, *left panel*), which coincidentally match with the nine node clusters identified earlier (Figure 1A). This insinuates that the SARS2 FLC structural pattern identified in each node cluster is a unique structural feature for the node cluster. However, we emphasize that the SARS2 FLC might not be the only factor determining the nine node clusters. In this regard and as possible future works, additional experimental evidence is needed to further prove the presence of the nine SARS2 FLC structural patterns from the nine nodal clusters, and, equally important, it would be interesting to likewise determine other factors that may explain the presence of the nine node clusters. Subsequently, we observed that no structural changes occurred in the SARS2 RBD (Figures 3B–J, *right panel*). In all the superimpositions made, no significant structural changes (RMSD < 1.0; Tm align > 0.96) occurred between superimposed SARS2 models (Figures 3B–J, *lower panel*), which is consistent with SARS2 maintaining its genomic integrity across propagation (34).

It was previously reported that the SARS2 FLC naturally undergoes polymorphisms, which in-turn affects viral transmissibility and tropism (39). In this regard, we suspect that the putative nine SARS2 FLC structural patterns are a product of natural polymorphism and, similarly, finding one of the SARS2 FLC structural patterns in one of the node clusters identified could suggest that certain countries (or continents) with overlapping node clusters may have varying levels of viral transmissibility and virulence (33, 34, 39). Since cleavage of the SARS2 FLC is a prerequisite for pathogenesis (17–19), we think that cleavage among the nine SARS2 FLC structural patterns may likewise vary (possibly depending on how exposed the FLC is), which in turn, could directly affect viral transmissibility. Additionally, with regards to host tropism, there seems to be no noticeable structural change in the SARS2 RBD, insinuating that host tropism is unchanged. This indicates that, regardless of any structural variations in SARS2 FLC, host tropism will not be consistently affected by genomic integrity (34). However, it is unclear whether the absence of SARS2 FLC (particularly ^681^PRRA^684^) would affect SARS2 RBD.

### SARS2 RBD Residues Did Not Change in the Absence of the Unique ^681^PRRA^684^ Sequence

**Figure 4:**
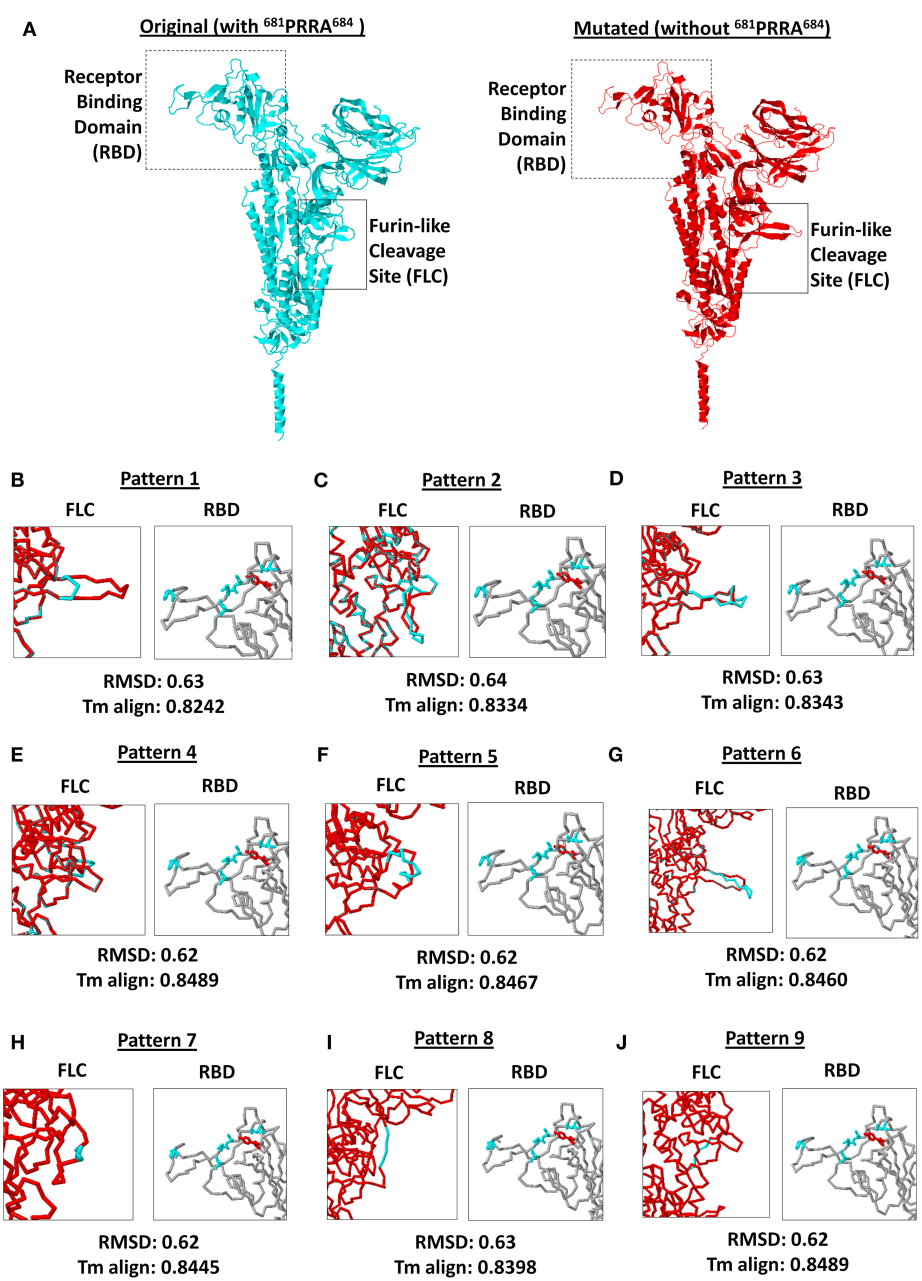
Comparison between original (with ^681^PRRA^684^) and mutated (without ^681^PRRA^684^) forms of the 9 SARS-CoV-2 spike protein furin-like cleavage site structural patterns and corresponding receptor binding domains. **(A)** Original (cyan) and mutated (red) representative monomeric SARS-CoV-2 spike proteins are shown. Receptor binding domain (boxed dash lines) and furin-like cleavage site (boxed solid lines) indicated. **(B–J)** Superimposed spike protein models showing the 9 structural patterns of the furin-like cleavage site (*left panel*) and receptor binding domain (*right panel*). Original (cyan) and mutated (red) SARS-CoV-2 spike protein furin-like cleavage site structural patterns and corresponding receptor binding domains are shown. RMSD scores and Tm align values normalized to the original SARS-CoV-2 spike protein model are indicated below.

SARS2 has been reported to infect multiple species as well as humans due to variations in ACE2 receptors across species (40), which emphasizes the potential significance of the SARS2 RBD with regards to host tropism. Similarly, SARS2 FLC was found to likewise affect host tropism (17–19). This may suggest that SARS2 FLC (particularly ^681^PRRA^684^) could affect SARS2 RBD. To establish the possible structural influence of the unique ^681^PRRA^684^ amino acid sequence on SARS2 RBD structural orientation, we generated mutant SARS2 models with the unique ^681^PRRA^684^ amino acid sequence deleted in all nine SARS2 FLC structural patterns and, subsequently, superimposed each mutant to the original model for comparison. This study undertook a side-by-side comparison of an original (*left panel*) and mutant (*right panel*) SARS2 model with a focus on SARS2 RBD (box dash lines) and FLC (box solid lines) structural changes (Figure 4A). As expected, in the absence of the ^681^PRRA^684^ amino acid sequence we observed structural variations in the SARS2 FLC (Figures 4B–J, *left panel*). Nevertheless, no significant structural changes were observed (RMSD < 1.0; Tm align > 0.82) between superimposed original and mutated SARS2 models (Figures 4B–J, *lower panel*). Most surprisingly, no structural variations were observed in the SARS2 RBD (Figures 4B–J, *right panel*). This would suggest that SARS2 FLC (particularly ^681^PRRA^684^) has no structural influence on SARS2 RBD, which is consistent with earlier works (41) that showed that SARS2 FLC may not be as critical as previously thought for the high fusion capacity of SARS2. However, it is worth mentioning that regions with high levels of the disorder typically do not have stable structures, and thus, would not have much of an effect on the remaining structured parts of the protein (20) consistent with our observations. Taken together, the lack of a stable structure in the FLC site and its surroundings may explain why no structural changes occurred within the SARS2 RBD after the removal of a unique ^681^PRRA^684^ region. Nevertheless, we presume that regardless of the absence of any structural variations within the SARS2 RBD, viral pathogenesis was unaffected since one important factor that determines virulence is high-affinity virus receptor interaction and, likewise, takes into account multiple host factors (40). This may explain why SARS2 infection in humans varies among COVID-19 infected patients. Additional experiments are needed to further prove this point.

## Discussion

SARS2 FLC is a conserved structural feature that is crucial for viral entry to host cells (39, 42) and, more importantly, can influence viral pathogenesis and host tropism (17–19, 40). In addition, the SARS2 FLC was found to have a naturally occurring polymorphism that can affect both transmissibility and host tropism (39). Throughout this study, we attempted to show that the SARS2 FLC has structural orientation variations putatively associated with the SARS2 genomic distribution particularly between December 2019 and July 2020.

SARS2 genome has continued to mutate since its emergence in December 2019 and SARS2 was found to have a >7.23 actual mutation rate with genetic changes occurring every other week (33, 34). These mutational changes are made possible through host-dependent RNA editing associated with the APOBEC mechanism (43). Cluster infections have also been associated with SARS2 incubation period infection and, likewise, play an important role in the rapid evolution of COVID-19 transmission (44, 45). This highlights how quickly the SARS2 genome is changing and, similarly, may explain how multiple variants of the virus can evolve easily and spread worldwide (33, 34). Several of the SARS2 nucleotide changes are nonsynonymous, thus, amino acid changes likewise occur (33) that may result in protein structural changes among SARS2 viral proteins. In particular, several structural changes have been reported with regards to the SARS2 spike protein (39, 42, 46, 47). Considering that we observed nine SARS2 FLC structural patterns from nine node clusters distributed worldwide, we postulate that this observation is putatively correlated to mutational changes that occurred within the SARS2 spike genome during the timeframe studied which in-turn affected the resulting amino acid sequence and, subsequently, lead to structural changes that may affect virulence and tropism.

It is worth mentioning that COVID-19 symptoms vary in the human population and, similarly, animal species (40). SARS2 infection in the human population often affects the lower respiratory tract (48) and follows a distinguishable order of symptom onset with varying levels of severity (49–51). COVID-19 reinfection has been clinically observed (52–56) and we suspect it is associated with varying SARS2 variants. In this regard, we hypothesize that COVID-19 reinfection could potentially be linked to SARS2 FLC structural variations since SARS2 FLC affects viral pathogenesis, tropism, and transmissibility. Admittedly, additional experiments are needed to further prove this hypothesis.

In summary, we propose that between December 2019 and July 2020, nine SARS2 FLC structural patterns could putatively correspond to the nine node clusters found within the COVID-19 genomic epidemiology network. Similarly, we associated this with the rapid evolution of the SARS2 genome. We observed that either in the presence or absence of the unique ^681^PRRA^684^ amino acid sequence no structural changes occurred within the SARS2 RBD, which we believe could mean that the SARS2 FLC has no structural influence on SARS2 RBD and may explain why host tropism was maintained.

## Data Availability Statement

The raw data supporting the conclusions of this article will be made available by the authors, without undue reservation.

## Author Contributions

MC and KI conceptualized the idea, provided feedback, helped in both structural and network analyses, and wrote the paper. MU, RI, and TH generated the protein models and analyzed the structural changes. YM, KY, NK, KW, and KB designed and analyzed the network. All authors contributed to the article and approved the submitted version.

## Conflict of Interest

The authors declare that the research was conducted in the absence of any commercial or financial relationships that could be construed as a potential conflict of interest.
