# Supplementary material for: Insights on the Structural Variations of the Furin-Like Cleavage Site Found Among the December 2019–July 2020 SARS-CoV-2 Spike Glycoprotein: A Computational Study Linking Viral Evolution and Infection
Source: Front Med (Lausanne). 2021 Mar 10;8:613412. doi: 10.3389/fmed.2021.613412 (PMC7987684; doi:10.3389/fmed.2021.613412)
Supplement: Supplementary file 1 [file Presentation_1.PPTX]

## Slide 1
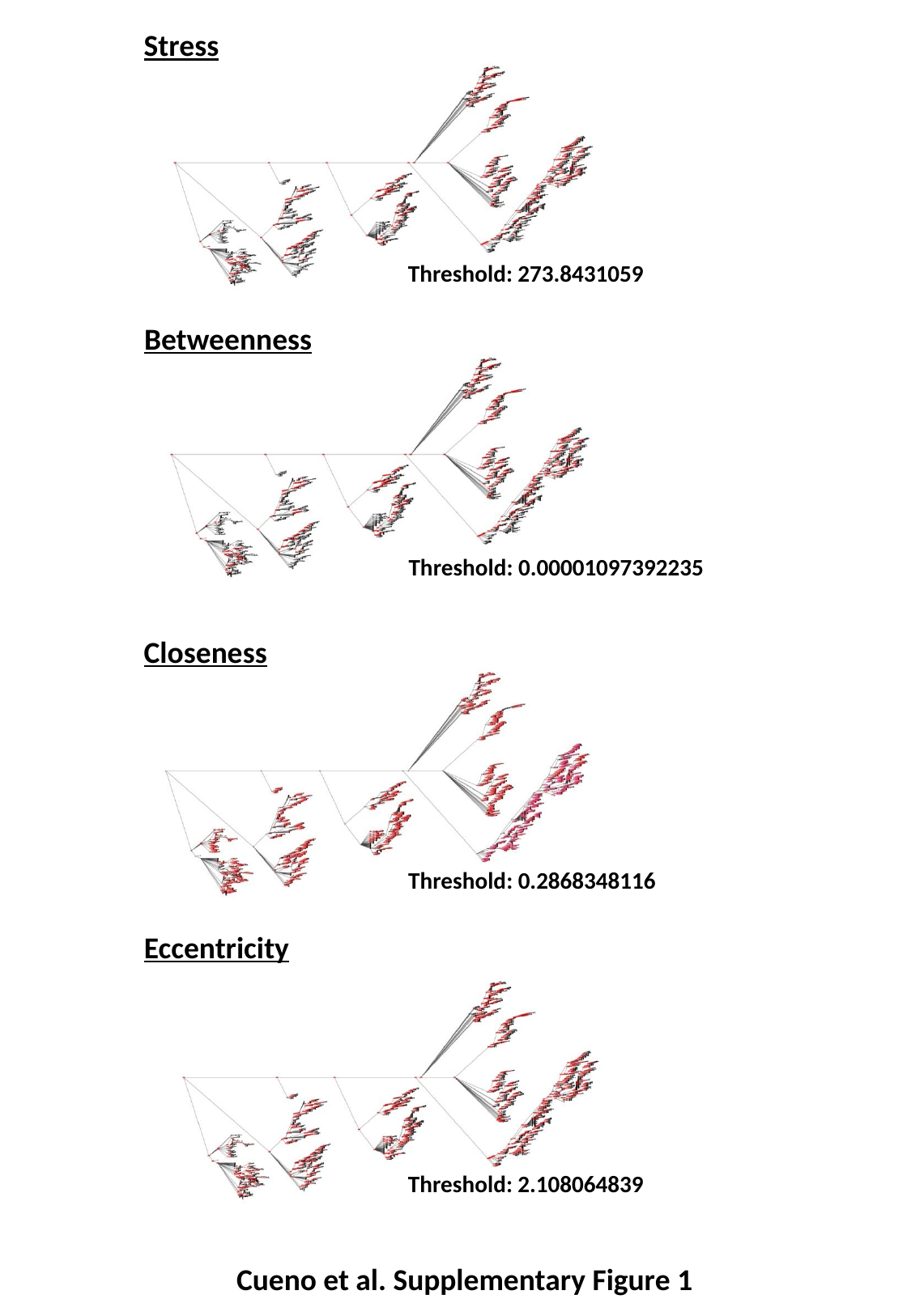

Stress
Threshold: 273.8431059
Betweenness
Threshold: 0.00001097392235
Closeness
Threshold: 0.2868348116
Eccentricity
Threshold: 2.108064839
Cueno et al. Supplementary Figure 1

## Slide 2
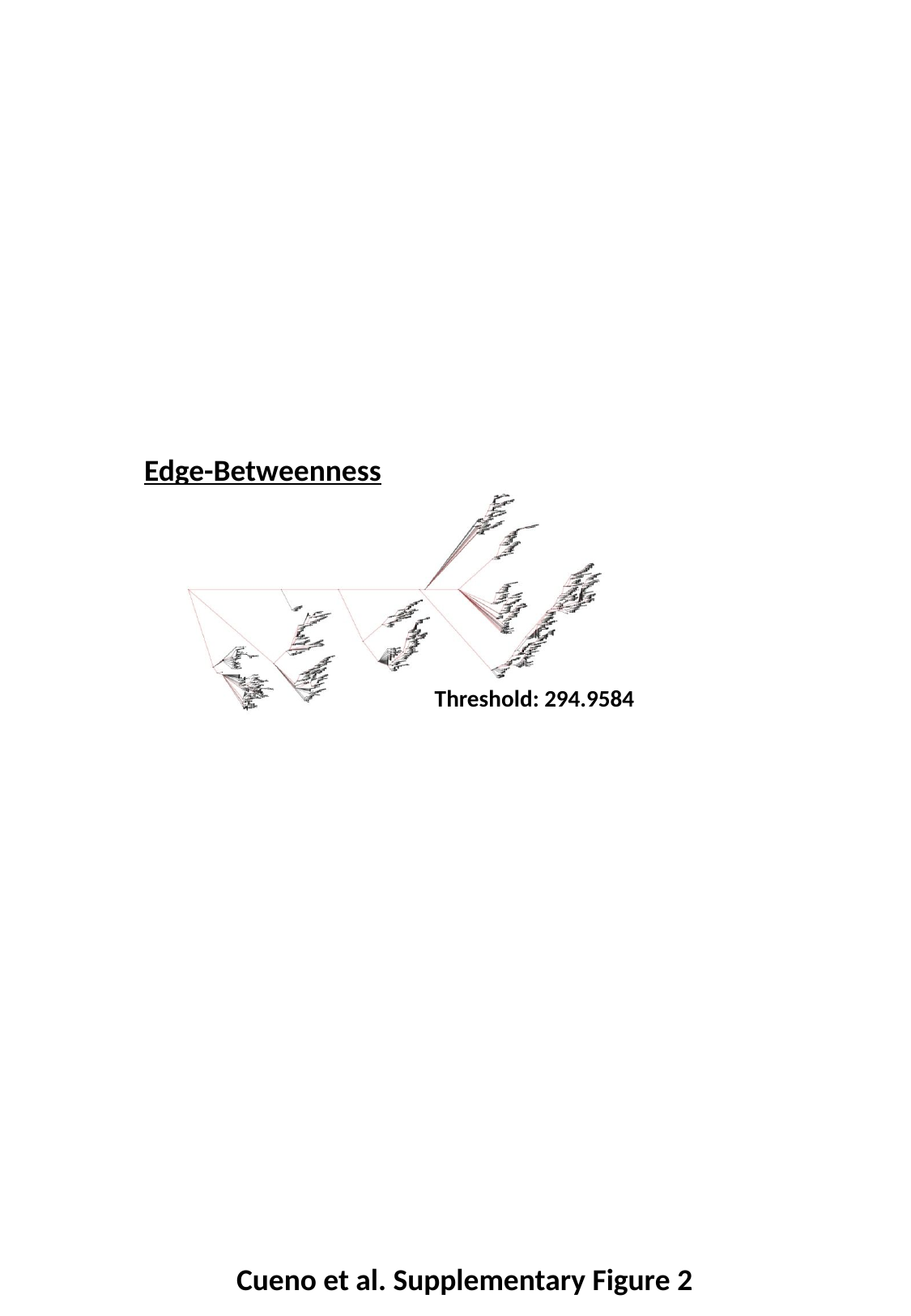

Edge-Betweenness
Threshold: 294.9584
Cueno et al. Supplementary Figure 2
